# Targeting Strategies for the Combination Treatment of Cancer Using Drug Delivery Systems

**DOI:** 10.3390/pharmaceutics9040046

**Published:** 2017-10-14

**Authors:** Janel Kydd, Rahul Jadia, Praveena Velpurisiva, Aniket Gad, Shailee Paliwal, Prakash Rai

**Affiliations:** 1 Department of Biomedical Engineering and Biotechnology, University of Massachusetts, 1 University Ave, Lowell, MA 01854, USA; janel_kydd@student.uml.edu (J.K.); Rahul_Jadia@student.uml.edu (R.J.); Praveena_Velpuri@student.uml.edu (P.V.); 2 Confocal Imaging Core, Beth Israel Deaconess Medical Center, 330 Brookline Avenue Boston, MA 02215, USA; Aniket_Gad@student.uml.edu; 3 Department of Chemical Engineering, University of Massachusetts, 1 University Ave, Lowell, MA 01854, USA; Shailee_Paliwal@student.uml.edu

**Keywords:** tumor targeting, nanomedicine, drug delivery, multidrug resistance, cellular, vascular, tissue, combination treatment, enhanced permeability and retention (EPR) effect

## Abstract

Cancer cells have characteristics of acquired and intrinsic resistances to chemotherapy treatment—due to the hostile tumor microenvironment—that create a significant challenge for effective therapeutic regimens. Multidrug resistance, collateral toxicity to normal cells, and detrimental systemic side effects present significant obstacles, necessitating alternative and safer treatment strategies. Traditional administration of chemotherapeutics has demonstrated minimal success due to the non-specificity of action, uptake and rapid clearance by the immune system, and subsequent metabolic alteration and poor tumor penetration. Nanomedicine can provide a more effective approach to targeting cancer by focusing on the vascular, tissue, and cellular characteristics that are unique to solid tumors. Targeted methods of treatment using nanoparticles can decrease the likelihood of resistant clonal populations of cancerous cells. Dual encapsulation of chemotherapeutic drug allows simultaneous targeting of more than one characteristic of the tumor. Several first-generation, non-targeted nanomedicines have received clinical approval starting with Doxil^®^ in 1995. However, more than two decades later, second-generation or targeted nanomedicines have yet to be approved for treatment despite promising results in pre-clinical studies. This review highlights recent studies using targeted nanoparticles for cancer treatment focusing on approaches that target either the tumor vasculature (referred to as ‘vascular targeting’), the tumor microenvironment (‘tissue targeting’) or the individual cancer cells (‘cellular targeting’). Recent studies combining these different targeting methods are also discussed in this review. Finally, this review summarizes some of the reasons for the lack of clinical success in the field of targeted nanomedicines.

## 1. Introduction

Cancer is ranked as one of the leading causes of death—second only to heart disease—and represents a major worldwide health concern [1]. In 2016, over 1.6 million new cases were projected to occur in the United States alone, along with over 500,000 cancer related deaths [1]. While better diagnostic, preventive and treatment measures have certainly helped to decrease incidence rates for some cancers such as those of colorectum and prostate, death rates from cancers of the liver, pancreas, and uterine corpus are still increasing despite progress in treatment methods [1]. A growing understanding of oncogenes and tumor suppressor genes has allowed us to develop newer technologies and conduct further research on the most efficacious ways to treat cancer [1,2,3]. The primary and most efficient form of cancer treatment consists of surgical resection of tumors followed by chemotherapy as a means of improving therapeutic efficacy and patient survival outcomes [4]. Imaging modalities, such as ultrasound, computed tomography (CT), magnetic resonance imaging (MRI), and positron emission tomography (PET) have played a crucial role in locating tumors and cancer metastasis in the body, which allow for improved implementation of treatments such as chemotherapy and radiation [5]. Novel treatment modalities like immunotherapy have also been recently approved for cancer treatment [5]. While these treatments have sometimes proven effective at treating cancer, they often have severe side effects that may be avoided with a more precise and targeted treatment, capable of providing localized drug payloads to tumor cells while rendering these drugs less harmful to normal cells [5].

Nanomedicine has developed in response to the need for drug delivery methods that resolve issues with poor drug solubility, nonspecific cytotoxicity, suboptimal pharmacokinetics and pharmacodynamics, as well as poor bioavailability [4,6,7,8]. Examples of drug delivery systems (DDS) include liposomes, polymeric nanoparticles, dendrimers, micelles, mesoporous silica nanoparticles and gold nanoparticles, among others [9,10]. Moreover, efforts have been made to enhance the therapeutic efficacy of several chemotherapy drugs by encapsulating them in exosomes, making them novel natural DDS [11,12]. The design characteristics of the nanoparticles are driven by the application of such DDSs, including surface charge and modification, shape, mechanical strength and chemical structure. These design parameters can be easily and conveniently altered, making nanomedicine an important tool in the treatment of cancer, as well as other diseases [9,10].

DDSs have been designed to accommodate both ‘active’ and ‘passive’ targeting of cancer [10,13,14,15,16]. Passive targeting is a means by which DDS can enter tumors due to enhanced fenestrations in tumor vasculature and take advantage of the enhanced permeability and retention (EPR) observed in solid tumors [17]. The EPR effect allows for some selective tumor uptake and retention of nanoparticles due to the leaky tumor vasculature and poor lymphatic drainage in tumors respectively [6,7,14]. Surface modifications of nanoparticles using polyethylene glycol (PEG), for example, can extend the circulation time of nanoparticles in the blood, while reducing the likelihood of the mononuclear phagocytic system (MPS) recognition and removal of the DDS [18,19,20,21]. Various examples of Food and Drug Administration (FDA) approved liposomal formulations include Doxil, a pegylated liposomal formulation of doxorubicin, Vyxeos, a liposomal combination of cytarabine and daunorubicin, and Onivyde, a liposomal formulation of irinotecan [17,22,23].

## 2. Targeted Nanomedicines

Surface modification of nanoparticles using specific ligand conjugation characterizes active targeting [10,21,24,25]. For example, specific cell surface receptors, such as transferrin, or folate receptors are overexpressed on cancer cells, including glioblastoma and breast cancer, among other types of cancer [26,27]. Nanoparticle surface modification with peptides, aptamers, monoclonal antibodies and small molecules which bind to the overexpressed receptor may increase cell-specific uptake via receptor mediated endocytosis (RME), whereby the DDS accumulates inside the target cell and delivers the drug payload [17,27,28,29,30,31]. Upon encountering the acidic environment of the endosome, a portion of the cell membrane which envelopes the DDS, transfers the DDS from the extracellular to intracellular domain, and finally, ligand-receptor complexes dissociate, releasing free receptors, which are recycled to the cellular plasma membrane [27,32].

Examples of the potential to improve cancer treatment using nanoparticle modifications include a study by Guo et al which used active targeting in vivo to compare transferrin-conjugated nanoparticles to their unconjugated counterpart [33]. The conjugated nanoparticles demonstrated better tumor growth inhibition than non-targeted nanoparticles [33]. Another study by Qin et al. implemented dual cyclic arginine-glycine-aspartic acid (RGD) peptide and transferrin conjugated nanoparticles, which not only targeted transferrin receptors but also penetrated the blood-brain barrier for glioma treatment [34]. Magnetic iron oxide nanoparticles have been used to eradicate tumors by magnetic hyperthermia as well as allow for imaging of the tumor, an additional application of nanomedicine [35,36]. Such nanoparticles have been surfaced modified with monoclonal antibodies such as Trastuzumab to target human breast cancers in vitro and in vivo [37]. Gold nanoparticles have been surface modified with biotin for targeting due to the increased cleavage of biotin by glutathione—a reducing agent—which is in high concentrations in cancerous cells. In vivo studies showed a marked 3.8-fold reduction in tumor volume when the biotinylated nanoparticles were administered in a HeLa cell xenograft tumor model [38].

**Figure 1:**
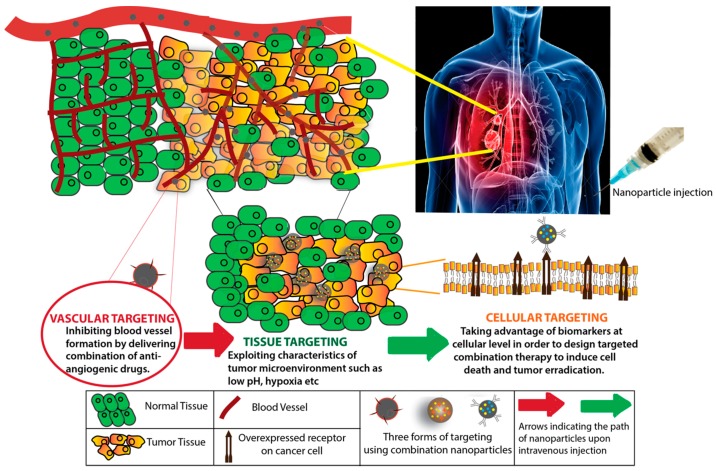
Schematic representation showing various levels of combination targeting using nanoparticles.

Various strategies have been employed to selectively destroy tumors including going after the tumor vasculature, targeting the tumor tissue (or tumor microenvironment), as well as cancer cell-specific targeting with nanoparticles or combinations of two or more of these approaches. Each of these approaches to targeting solid cancers have specific considerations in terms of choice of biological target and nanoparticle design parameters, specifically the surface functionalization with appropriate targeting ligands. Typically, anti-cancer nanomedicines are administered in patients via an intravenous infusion and these nanoparticles have to overcome several biological barriers as they traverse through the body from the injection site to the site of action inside the body. The path that these drugs follow in the body after injection include circulating in the vascular network, reaching the tumor vasculature followed by traversing through the large fenestrations in the vasculature into the tumor tissue microenvironment. Some of the trapped drugs do leak out of the nanoparticles all along this path and can either passively diffuse across cell membranes or can be internalized via receptor mediated endocytosis (RME). The receptor-mediated internalization brings the encapsulated drug to the cancer cells for intracellular action that eventually causes cancer cell death. This path following intravenous infusion of nanomedicine and the avenues for targeting cancer it provides are illustrated in Figure 1.

Targeting tumor vasculature is one method for targeting cancer and has been explored by several groups. By directing therapy towards receptors such as the integrin receptors or vascular endothelial growth factor (VEGF) receptors that promotes angiogenesis, new vasculature growth can be prevented, thereby removing an essential method by which the tumor receives nourishment [39,40,41,42]. Hu et al. found that overexpression of a certain micro RNA that is downregulated in non-small cell lung cancer cells could decrease the expression of VEGF and significantly decrease angiogenesis [43]. Cancerous vascularization can also be inhibited through other means, such as the endoglin receptor, as shown by Toi et al. in 2014 [44]. While vascular level targeting has shown promise, uncontrolled factors in mouse models such as diet, sex, and genetic background have been unaccounted for in clinical trials and may be the reason why vascular level targeting has not been as successful in patients as other methods [42,45]. This of course highlights the need for better cancer models which are more representative of human disease and its variability.

Targeting the tumor’s microenvironment—at the tissue level—has proven effective in reducing tumor size, along with preventing pro-cancer behaviors such as angiogenesis. Tumor tissue in solid cancers typically exhibit an acidic pH relative to non-cancerous tissue due to the build-up of lactic acid on account of the “Warburg effect” [46]. This difference in pH has been exploited for tumor tissue targeting by several groups [47,48]. The microenvironment and physical characteristics such as increased interstitial pressure, as well as reduced nutrient and oxygen availability of tumor cells, can be harnessed as potential areas of exploitation by therapeutic methods described previously [7]. Specific to cell functioning and signaling there exists a further, more intricate approach of interfering with cellular processes, characteristic of cellular level targeting, a third way of targeting [49].

Cellular level targeting inhibits pro-cancer cellular pathways by binding targeting cell receptors and cytokines [49,50,51]. One particular study highlighting cellular targeting treated late stage gastric cancer by targeting the IL-6 cytokine, which binds to the IL-6R receptor and triggers a cascade response that promotes growth and inflammation [52]. By circulating a soluble form of the IL-6R receptor through the bloodstream to bind to free IL-6 cytokines, promotion of growth and inflammation in cancer cells was prevented [52]. In another study, Jin et al. developed a remotely triggered system that released 5-fluorouracil, which blocks enzyme activity that is essential for DNA replication [53]. Photodynamic therapy, a remotely-triggered treatment modality can be used to create a toxic environment in cancer cells, as shown in one study in which selenium-rubyrin particles were activated by near-infrared (NIR) light and caused reactive oxygen species to be generated, which produces irreparable damage to the cancer cells [54]. Induced hypoxia was also believed to be a suitable method for in vitro cancer cell death, as shown by Steinbach et al. [55]. Recent studies—such as Ammirante et al.—show that tissue injury and hypoxia may promote cancer progression, however, rendering a single targeting approach ineffective, encouraging the use of combinational therapy methods [56]. Several studies have shown that irradiated nanoparticles can produce a hyperthermic effect on cancer cells, including a study where rod-shaped gold nanocrystals were used to conduct photothermal therapy (PTT) on small cell lung cancer [57,58].

A significant hurdle encountered in cancer treatment is the development of tumor cell resistance to chemotherapeutic drugs [59,60,61,62]. Combining the targeted methods of treatment and using nanoparticles as DDSs decreases the likelihood that resistant clonal populations of cancerous cells will propagate by attacking cells with different effector routes [63,64]. The use of more than one chemotherapeutic drug allows simultaneous targeting of more than one characteristic of the tumor [65]. Targeted combination therapy also allows for a lower dosage of the drugs to reduce cytotoxicity while maintaining treatment efficacy by inducing synergistic killing and by targeting nanoparticles directly to cancer sites to avoid death of healthy cells [4,9,17]. In a study conducted in 2014, curcumin and a platinum drug, cisplatin, which target various parts of the cell’s internal functions and lead to apoptosis, were co-delivered in polymeric micelles and enhanced cytotoxicity to a cell line that was resistant to the platinum drug alone [61]. In another study, Yuan et al. showed that synergistically delivering ibuprofen and doxorubicin (DOX) preventing inflammation, which promotes pathways such as proliferation and differentiation of cancer cells, and disrupting the cell’s mechanism for replicating DNA [66]. A variety of different methods of combination therapy have proven to be effective at combating cancer resistance to chemotherapeutics and decreasing cytotoxicity to healthy cells [6,7,10,63,66]. When administered by a nanoparticle DDS, as opposed to free form injection of the drugs, combination therapy is even more efficient and less detrimental to healthy cells [6,67,68].

This review discusses recent studies that support the use of nanoparticles and combination treatment for various tiers of tumor targeting—vascular, tissue, and cellular (as shown in Figure 1). Recent studies describing progress made in the field of targeted nanomedicine towards the goal of improving cancer treatment methods and, in effect, the possible applications of these combinatorial DDSs in clinical trials for cancer therapy, are discussed. Finally, some potential reasons for the lack of clinical success in the field of targeted nanomedicines are also discussed.

## 3. Targeting Tumor Vasculature

Angiogenesis is critical in the transition of tumors from benign to malignant disease states [69]. The “angiogenic switch” allows for blood vessel growth from pre-existing vessels [6,70]. Blood vessel growth is critical for solid state tumor expansion in the body for tumor cells to adapt to the increasing nutrient and oxygen demands of mutated cancer cells, as well as the low pH conditions and increased interstitial pressure found in the tumor cell environment [45,71,72]. Hypoxia causes transcription of cellular hypoxia inducible factor (HIF), which in turn increases proangiogenic proteins, such vascular endothelial growth factor (VEGF), platelet derived growth factor (PDGF) and tumor necrosis factor-α (TNF-α) [42]. Replication of in vitro studies of therapeutic anticancer agents in vivo are plagued by the heterogeneous nature of the tumor microenvironment and subsequent failures in effective treatments [26]. In vivo barriers such as the vascular endothelium interfere with intravenous chemotherapeutics by reducing the permeability and direct cellular effects [69]. The endothelial cells which line blood vessels are key targets in disease processes such as cancer, as well as inflammation, ischemia, and thrombosis, among others [73]. Endothelial cell surface markers such as peptidases and cell adhesion molecules (CAM) are key targets for anticancer therapies [69]. Nanoparticles can bind to these cell surface markers and allow for cell membrane penetration and release of the chemotherapeutic payload encapsulated in the DDS [69]. The endothelium is an advantageous site of targeting due to its increased accessibility compared to circulating bodies such as tumor cells [69].

A major challenge associated with tumor vascular directed therapies is the risk of serious side effects in traditional intravenous chemotherapy treatment [29,31,74]. The synthesis and testing of more effective and less toxic nanomedicines have been investigated in vitro and in vivo, as well as in clinical trials. Humanized monoclonal antibodies, such as bevacizumab, matrix metalloprotease inhibitors, and small molecule tyrosine kinase inhibitors are examples of compounds used to target angiogenesis [75]. For example, bevacizumab free drug has implications such as poor patient compliance due to the dosage frequency and drug resistance, however in the encapsulated form, there is sustained slow delivery of the drug and increased time between administrations, making the nanoparticle form more desirable [76]. Bevacizumab has also demonstrated promising synergistic response when combined with CRXL101, the nanoparticle form of camptothecin, in preclinical models [77,78]. CRLX101 in the nano form has a half-life of nearly 24 h, compared to a mere 2 h as a free drug, resulting in prolonged drug exposure and further emphasizing the significance of encapsulating drugs [78]. As a monotherapy treatment for platinum-resistant ovarian cancer in preclinical studies, CRLX101 is effective at maximum tolerated dosages, however frequent low-dose CRLX101 given in combination with bevacizumab yielded superior tumor reduction and minimal toxicity when compared to both drugs given as monotherapies [77].

Vascular targeting has the advantage of acting upon the tumor microenvironment by interfering with cancer cell angiogenesis by inhibiting vascular endothelial growth factor receptor and targeting αvβ3 integrin by RGD peptides, an amino acid sequence consisting of Arg-Gly-Asp [79]. Integrins are cell adhesion receptors that bind and activate matrix metalloproteases (MMP-2), regulate cell attachment, spreading and migration [80]. Ligated αvβ3 integrins prevent apoptosis in cells and are integral in the process of angiogenesis. RGD-based sequences conjugated to the surface of nanoparticles can target and bind to αvβ3 integrins [26]. RGD conjugated nanoparticles are unique because they target in a dual manner whereby first pass is endothelial cells followed by subsequent extravasation and uptake by tumor cells as a secondary pass, thus enhancing the delivery of drugs into the tumor [81].

A study by Murugan used polyacrylic acid chitosan surface-modified mesoporous silica nanoparticle (MSN) to deliver topotecan (TPT) and querceptin (QT) to triple negative breast cancer cells (TNBC)(MDA-MB-231) and multidrug resistant breast cancer cells (MCF-7) [65]. RGD-peptide was grafted to the surface of the nanoparticles in order to target the αvβ3 integrin. In vitro and in vivo studies were carried out to assess cellular uptake and viability [65]. Both cellular uptake by cancer cells and release of encapsulated drugs were enhanced by the RGD-peptide, via integrin receptor mediated endocytosis and the acidic pH of the intracellular environment, respectively [65]. Molecular and structural changes of cellular endoplasmic reticulum, nucleus and mitochondria, as well as synergistic antiproliferative effects and cell death, were observed in both cell lines. MDA-MB-231 cells had higher cytotoxicity effects, approximately 88% cell death, while MCF-7 cells had 63% cell death [65]. The difference suggested that receptor mediated endocytosis in the overexpressed integrin receptor may have caused the varied cytotoxic effects between cell lines [65].

Albumin-based nanoparticles targeting the αvβ3 integrin receptor, combined with photodynamic therapy (PDT), can improve the therapeutic efficacy of anticancer drugs when compared to conventional monotherapy [39,82,83]. Tumor-targeted multifunctional albumin-based nanoparticles prepared by drug-induced self-assembly were used in a study by Chen et al. to treat U87 human glioblastoma cells in vitro and in vivo [84]. Albumin-based nanoparticles are biocompatible, abundant and provide an alternative method to drug delivery [82,85,86]. Paclitaxel, a chemotherapy drug, binds to human serum albumin (HSA) and causes aggregation and self-assembly of nanoparticles [87]. This study used a photosensitizer, chlorin e6 (Ce6), which is a chelating agent for manganese-II, Mn^2+^, that enables magnetic resonance imaging (MRI), and acyclic Arg-Gly-Asp (cRGDyK) peptide, a targeting agent for 𝛼v𝛽3 integrin, which is overexpressed on tumor angiogenic endothelium. Two types of tumor-targeting theranostics were designed by (1) simultaneous coassembly of HSA-Ce6 and HSA-RGD forming HSA-Ce6-PTX-RGD-1 and (2) formation of HSA-Ce6@HSA-RGD core-shell structure, or HSA-Ce6-PTX-RGD-2. Nanoparticles were formed using Paclitaxel (PTX) to cause self-assembly by albumin aggregation, resulting from the hydrophobic interactions between PTX and the hydrophobic domain of HSA. Synergistic killing of cells using PDT by accelerated endosomal escape of drugs and Mn^2+^ MRI tracking were both utilized in this study [87].

**Figure 2:**
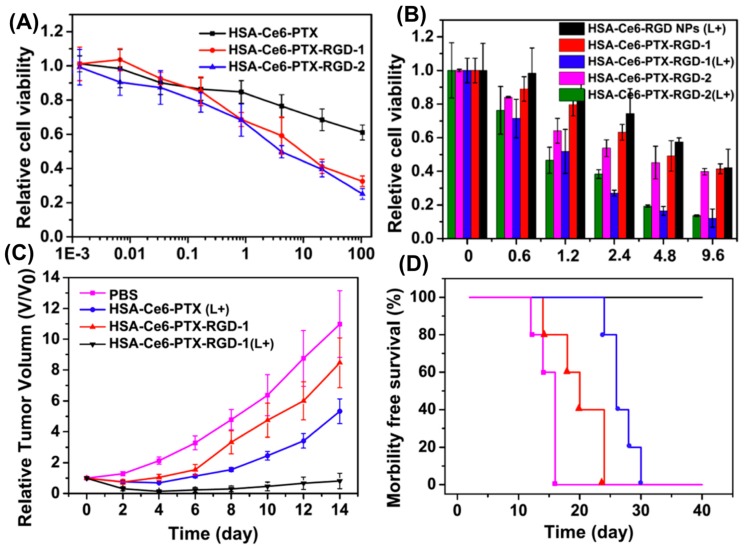
In vitro results for relative cell viabilities in (**A**) U87 cells treated with various formulations after 30 min incubation, followed by washing and reincubation in free medium for 48 h, followed by 3-(4,5-dimethylthiazol-2-yl)-2,5-diphenyltetrazolium bromide, MTT, assay, and (**B**) cell viability related to exposure to light irradiation at 660 nm (2 mW/cm^2^, 0.5 h), whereby photodynamic therapy was followed by rinsing in PBS and reincubation for 24 h, followed by the MTT assay. In vivo results for (**C**) and (**D**) tumor volume and morbidity free survival, respectively, comparing treatment groups with or without light irradiation and RGD peptide. The treatment group which had both light irradiation and incubation with nanoparticles encapsulating both ceramide and paclitaxel with RGD peptide surface modification displayed longest morbidity free survival and smallest tumor volume. Reproduced with permission from [87], Copyright ACS, 2015.

U87 cells were incubated with HSA-Ce6-PTX, HSA-Ce6-PTX-RGD-1, or HSA-Ce6-PTX-RGD-2. Confocal imaging and flow cytometry showed more fluorescence with Ce6 in RGD-1 and RGD-2 compared to HSA-Ce6-PTX. RGD-1 and RGD-2 demonstrated effective molecular targeting of the 𝛼v𝛽3 integrin overexpressed on tumor cells, as shown in Figure 2. The cytotoxicity of PTX was the same in free PTX, HSA-PTX, and HSA-Ce6-PTX, therefore the chemotherapeutic efficacy of PTX was not affected in the experiments. RGD-1 and 2 both showed significant increased killing due to the specific recognition of 𝛼v𝛽3 integrin by RGD. There was also a synergistic effect found when cells were treated with PDT and chemotherapy compared to PDT or chemotherapy alone. The specific binding of RGD nanoparticles to tumor cells was validated by higher tumor accumulation of RGD nanoparticles when compared to HSA-Ce6-PTX without RGD and Ce6. Ex vivo fluorescence imaging intensities for RGD nanoparticles were also 2.4 times higher. HSA-Ce6-PTX-RGD-1 with chemotherapy and no light exposure yielded short-term growth inhibition of cells, while addition of 660 nm light therapy caused complete inhibition. Mice treated with combination tumor-targeting survived 40 days, while other treatment groups lived 15–30 days [87].

The dual modeling imaging capabilities of this study in addition to the biocompatibility of albumin-based, tumor-targeted nanoparticles is an example of how chemotherapy and PDT synergism, along with visualization of treatment effect, can improve the success of cancer cell targeting and localized treatment [87].

Vascular level targeting is advantageous due to the extent of distribution of nanoparticles in the bloodstream and the bioavailability of targeted nanoparticles which have characteristics of extended circulation time and evasion of the RES and macrophages. The large distribution of vascular targeted therapy comes with the cost that nanoparticles may not reach the cancer tissue cells effectively. The inhibition of blood vessel growth and normal wound healing can also present dangerous health impairments to patients on such treatments. The conditions of the tumor microenvironment, or tissue level, are more advantageous as nanoparticles can be more cancer specific and penetrate the tumor for improved drug payload delivery.

## 4. Targeting the Tumor Microenvironment

One of the limitations of the existing treatments is the inability of the drugs to reach the deeper layers of tissue [88]. A few distinctive characteristics observed in cancerous cells such as low pH, enhanced glycolysis can be utilized in detection of tumors or delivering drugs to the tumor sites. pH is used as a marker to detect malignant cells and pH on the surface of tumor cells is lower than extracellular pH in healthy and tumor tissue [89,90,91].

Targeting via acidity-triggered nanoparticles in tumor tissue, Tapmeier et al. designed a detection system using pH low insertion peptides (pHLIPs) tagged with fluorescent Alexa546, that were found to be accumulated in tumors of pH less than 6.7 in 4T1 breast cancer cells implanted in BALB/mice [89,92,93]. With the rise in hydrophobicity of carboxyl groups, pHLIPs become protonated in low pH conditions causing insertion of peptide into the cell membrane [89,94]. On the other hand, Yu H. et al. investigated a combinatorial approach to deliver drugs to the tumor sites with low pH in 4T1 breast cancer model. Triple-layered micelleplex was used to deliver hydrophobic cisplatin and siRNA [95]. pH sensitive triblock polymer, PEG-b-PAGA-b-PDPA was chosen as the carrier where, in acidic environment, poly(2-(diisopropylamino)ethyl methacrylate), PDPA dissociates due to protonation of tertiary amine and releases the cargo at pH < 6.3 [95].

A study by Wu et al. showed that 84.94% of methotrexate (MTX) was released from Fe_3_O_4_MgAl-LDH (layered double hydroxide) nanoparticles of ~230 nm in the tumor with pH of 3.5 within 48 h. With the dosage of Fe_3_O_4_MgAl-LDH nanoparticles, higher antitumor activity was observed in HUVEC, MCF-7 and HepG2 cell lines [47]. In order to achieve a controlled release of the drug, research group of Wu J. et al. shielded Fe_3_O_4_@SiO_2_-DOX with chitosan (CS) [47]. These nanoparticles of 63 nm in size released 86.1% of DOX in pH conditions of 4.0 over 48 h where the release profile tracked Higuchi model. Significant antitumor activity was noticed in HepG2 cells [47].

Findings by Zhang H. et al. showed that conjugation of DOX to TiO_2_@Fe_3_O_4_/PEI nanoparticles via N-Fe-O coordination bond releases DOX at a rate of 86.4% at pH 5.2 while 15% at pH 7.4 over 24 h. This indicates the sensitivity of the coordination bond to lower pH conditions. A combination of this nano-formulation, along with laser irradiation, exhibited a tumor inhibition rate of 80% in S180 tumor (human liver cancer) mice models [96]. TiO_2_ is a safer nanoparticle with less toxicity in vitro and in vivo, while Fe_3_O_4_ is FDA approved nanomaterial with high biocompatibility. Fe_3_O_4_ used in the study increased the photo catalytic activity of TiO_2_ [47,96,97,98,99,100,101].

Another setback with the current chemotherapy that targets cancer tissue is multidrug resistance (MDR). Increased expression of P-glycoprotein (P-gp) promotes the drug efflux, thus leading to poor intracellular retention of the drug. In order to overcome MDR, strategies employing nanoparticles (NPs) of suitable size and shape that can be retained in the tumor have been designed.

Chen et al. created nano drug delivery systems that release the drug in the tumor micro environments after they undergo a physiological change in their shape [102]. The spherical micelles in the presence of matrix metalloproteinases (MMPs) in the tumor transform into a nano-fiber causing increased accumulation of the drug in the tissue. These nanomicelles (HA-MSDOX-KLA) constitute hyaluronic acid (HA), MMP substrate conjugated to doxorubicin and a pro-apoptotic peptide (KLAKLAK)_2_, referred as KLA in the article [102].

**Figure 3:**
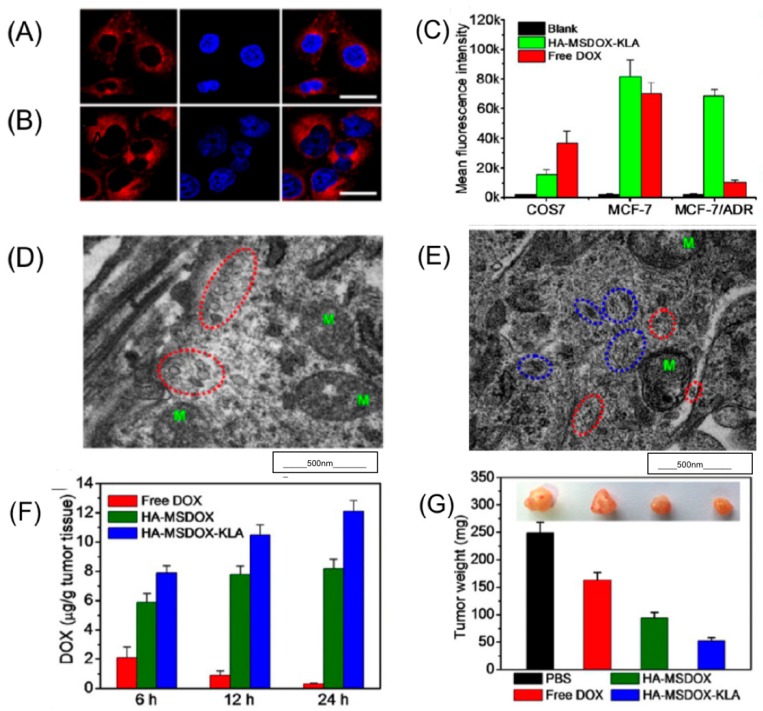
Confocal laser scanning microscopy (CLSM) images of (**A**) MCF-7 and (**B**) MCF-7/ADR cells treated with HA-MDSOX-KLA after 6hours. Scale bar: 30 µm. (**C**) Mean fluorescence intensity of DOX internalized by MCF-7 and MCF-7/ADR cells when treated with free DOX and HA-MDSOX-KLA. (**D**) Bio-TEM images of MCF-7/ADR treated with HA-MDSOX-KLA after 6 hours and (**E**) 24 h. M represents the mitochondria, the red circle showing the nanoparticles, while the blue circle shows the nanofibers. Scale: 500 nm. (**F**) Accumulation of DOX in tumor after intravenous administration of all formulations with 2 mg/kg of DOX. (**G**) Representation of tumor weights; each treated with different formulations. Reproduced with permission from [102], Copyright ACS, 2016.

The strategy of this study was as follows: MMP substrate in micellar NPs is cleaved when surrounded by higher levels of MMPs, thus increasing the hydrophobicity of the particles. Nanofibers are then formed and KLA peptide is released. HA-MSDOX-KLA micelles functionalized with HA, had a diameter of 38.2 ± 3.7 nm as observed in transmission electron microscopy (TEM). When exposed to MMPs, the nanofibers thus formed had a diameter of 30–40 nm and were 200–300 nm long. To understand the active tumor targeting these NPs were incubated with MCF-7 (breast adenocarcinoma), MCF-7/ADR (multidrug resistant breast adenocarcinoma) and Cos-7 cell lines for 6 h. Significant red fluorescence due to DOX was found in MCF-7/ADR and MCF-7 cell lines as shown in Figure 3A,B.

Mean fluorescence intensity (MFI), as shown in Figure 3C, indicates that the uptake of HA-MSDOX-KLA and free DOX was higher in MCF-7 cells while in MCF-7/ADR, higher uptake of HA-MSDOX-KLA and a significantly low amount of free DOX was seen. The low fluorescence intensity due to free DOX in MCF-7/ADR explains that the P-gp pumped out the free DOX while retaining the nanofibers. The higher uptake of HA-MSDOX-KLA by CD44+ tumor cells via receptor mediated endocytosis is facilitated by active targeting of HA peptide.

MFI of HA-MSDOX-KLA increased 6.5-fold in MCF-7/ADR cells after 6 h and a 9.6-fold increase was observed after 12 h compared to free DOX. Bio-TEM images of MCF-7/ADR cells further confirmed the uptake of the transformed and non-transformed NPs. As shown in Figure 3D, after 6 h of incubation, mitochondria were intact while the particles were micellar shaped. But after 24 h, the mitochondrial cristae were deformed and the nanoparticles assumed fibrous structures as shown in Figure 3E. This explains the phenomenon in which MSDOX was cleaved and released KLA peptide, which in turn triggered apoptosis leading to mitochondrial deformation degrading ATP levels. 3-(4,5-dimethylthiazol-2-yl)-2,5-diphenyl tetrazolium bromide (MTT) assay results demonstrate that MDR factor of HA-MSDOX-KLA was significantly lower than the free DOX confirming the anti-MDR effect of the nano formulation.

In vivo studies performed in MCF-7/ADR tumor bearing mice showed that higher DOX internalization was seen in HA-MDSOX-KLA treated mice compared to HA-MSDOX and free DOX dosed mice as shown in Figure 3F. Apoptosis and maximum number of non-dividing cells were observed in mice treated with HA-MSDOX-KLA compared to the other groups. Tumors from MCF-7/ADR mice were collected after 16 days of treatment. The tumor weights when measured indicate a significant decrease in the group treated with HA-MSDOX-KLA compared to others, as shown in Figure 3G. The tumor-triggered nano-formulation resulted in increased intracellular retention of the drug through its morphological transformation. This study provided an efficient method to combat drug resistant tumors [102].

## 5. Cellular Level Targeting

Expression of cell markers which are indicative of proliferating cells are a means by which cellular targeting can be utilized and visualized in vivo for diagnosis and staging. Particular cell markers, signaling pathways, cell surface receptors, such as folate, transferrin and epidermal growth factor receptor (EGFR), as well as stem cells, immune cells, stromal cells, and fibroblasts, may be used for targeting as they are either upregulated, downregulated or mutated in rapidly dividing cells during cancer [18,81,103,104,105,106]. Cellular targeting can be direct, indirect or combination as we discuss here using specific studies and conglomerations of works cited in literature reviews.

### 5.1. Direct Cellular Targeting

The higher level of specific receptors on tumor cells surface compared to normal cells make it plausible to design functionalized nanoparticles that can specifically bind to these overexpressed receptors. Folate and transferrin receptors stand out the most since several tumors show higher levels of folate and transferrin [24,27,107,108,109].

**Figure 4:**
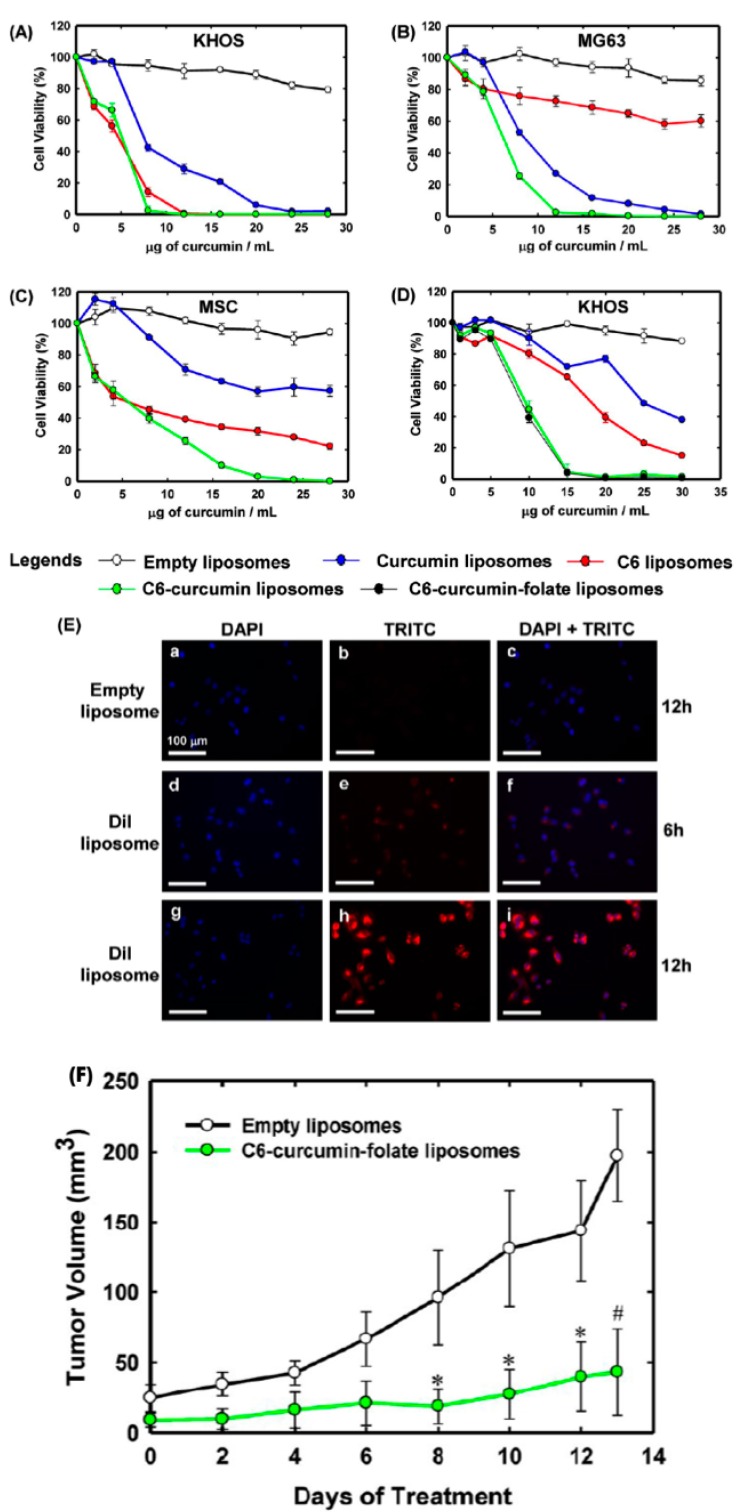
The results of cell viability testing in (**A**,**D**) KHOS, (**B**) MG-63 and (**C**) MSCs cell lines show that C6-curcumin liposomes are significantly more effective in killing cancer cells compared to other formulations tested, including single drug loaded liposomes and empty liposomes. Folate targeted C6-curcumin liposomes were also tested in (**D**), yielding a similar cell viability trend as non-targeted C6-curcumin liposomes. Liposomal uptake studies shown in (**E**) display fluorescence due to C6-curcumin over 6 and 12 h. Tumor volume was significantly decreased in (**F**) by treatment with C6-ceramide-folate liposomes. Reproduced with permission from [104], Copyright ACS, 2014.

A study by Dhule et al. discussed the combined effect of liposomal encapsulation of curcumin (Cur) and C6 ceramide on osteosarcoma (OS) cell lines, MG-63 and KHOS OS, and non-cancerous, untransformed primary human cells (human mesenchymal stem cells (MSCs)). Curcumin is a hydrophobic drug characterized by potent anticancer effects such as tumor initiation blockage, suppression of tumor progression, inhibition of invasion and metastasis by acting on vascular endothelial growth factor, cyclooxygenase, matrix metalloproteases, among others. C6 ceramide, a sphingolipid, is another anticancer agent which contributes to curcumin mediated cell death because of its role cell cycle arrest, apoptosis, growth inhibition and senescence. Modification and targeting of liposomes with polyethylene glycol (PEG) and folate (FA), respectively, is a desired drug delivery system (DDS) of hydrophobic compounds such as curcumin and C6 ceramide because of the longer plasma life of the drugs systemically and specific drug delivery to osteosarcoma cells which overexpress folate. OS, an extremely aggressive form of bone cancer, is characterized by high heterogeneity in the tumor cell environment which leads to challenges in treatment caused by variable antigenicity, chemo-sensitivity, growth rate and karyotype. The use of this combined drug therapy which provides targeted delivery is of significant clinical importance in effective treatments of OS [104]. The effects of curcumin, C6, and C6-Cur liposomes on MG-63, KHOS, and MSCs showed greater cytotoxicity in MG-63 cells when treated with C6-Cur liposomes compared to C6 and Cur liposomes. KHOS cells were 1.5 times more sensitive to C6-Cur and C6 liposomes compared to Cur liposomes. MSCs were resistant to Cur due to the characteristic of nonmalignant cells to arrest in G_0_ phase reversibly with no apoptosis occurring. MSCs showed a higher resistance to C6-Cur liposomes at increased concentrations, thus potentially providing better therapeutic ranges for treatments with less toxicity. Cell growth rate determines the uptake of liposomes into cells and subsequent drug efficacy. Cell cycle assays on KHOS cells showed that curcumin liposomes induced cell cycle arrest in the G2/M stage by increased upregulation of cyclin B1, while C6 liposomes induced G1 arrest by downregulating cyclin D1 and C6-Cur liposomes induced G2/M cell cycle arrest with combined effects on the expression levels of cyclins B1 and D1. In vivo testing using human xenograft osteosarcoma assays revealed significant decreases in tumor size with C6-Cur-FA liposome treatment, compared to other Cur and C6 liposomes [104]. The results of the study are presented in Figure 4 below.

The use of Cur for MDR reversal by decrease in the expression and function of P-glycoprotein, as well as induction of caspase dependent and independent apoptosis in combination with C6 ceramide provides a treatment for solid OS tumors which could overcome heterogeneous cellular resistance to drug efficacy [104]. Liposomal drug delivery of these anticancer compounds provides a means to protect water-insoluble drugs from enzyme degradation during systemic circulation and inside the cell. Improved drug efficacy and circulation time within the body with fewer side effects due to the targeted combination therapy as presented in this study further provides better treatment possibilities for drug resistant cancer types [110,111,112,113].

Another study used a folate analog, methotrexate (MTX) codelivered with mitomycin C (MMC) by conjugation to PEGylated chitosan nanoparticles [114]. MMC inhibits DNA replications by interfering with DNA synthesis and nuclear division, while MTX impedes nucleic acid biosynthesis by reacting with dihydrofolate reductase. Thus, MTX not only inhibits metabolism of folic acid but also enters cells through a similar mechanism as folic acid thereby targeting PEGylated chitosan nanoparticles to the over expressed folate receptors. MTX + MMC codelivery using chitosan nanoparticles showed synergistic killing efficacy when tested against monodelivery of MTX and MMC [114]. In another study, Lin et al. co-delivered MMC with 10-hydroxycamptothecin (HCPT) using folate functionalized soybean phosphatidylcholine micellar nanoformulation to test the therapeutic efficacy in HeLa Cells [115]. Direct cellular targeting of MMC and HCPT using micellar nanoparticles not only enhanced cellular uptake in in vitro and in vivo but also showed significant reduction in tumor burden compared to free drugs. There have been several other studies using folate targeted combination nanoparticles showing not only synergistic killing efficacy in vitro and in vivo but also improved biodistribution profile [107,108,116]. For example, a study conducted by Pawar and colleagues showed that folate functionalized solid lipid nanoparticles delivering curcumin and docetaxel had significant decrease in docetaxel accumulation in heart and kidney when compared to the approved Taxotere^®^ [117].

Targeted nanomedicines using transferrin ligand are extensively studied. In the study by Malarvizhi et al., doxorubicin and sorafenib were co-encapsulated in transferrin functionalized polyvinyl acetate core-albumin shell nanoparticle [118]. The nanoformulation showed synergistic killing efficacy (92%) when compared to free drugs (50%) and non-targeted core-shell nanoparticle (63%). Transferrin targeted PEGylated phosphatidylethanolamine micelles carrying curcumin and paclitaxel showed increased cytotoxicity in paclitaxel-resistant ovarian adenocarcinoma as shown by Sarisozen et al. [119]. Another approach used co-delivery of doxorubicin and curcumin in transferrin targeted “PEGylated curcumin” nanoparticles as shown by Cui et al. for the treatment of breast cancer [120]. This combination nanoparticle showed significant tumor regression in xenograft mice model when compared with curcumin/doxorubicin liposomal formulation and the single drug treatment [120].

Direct cellular targeting as mentioned previously is advantageous in terms of more specific drug delivery, however these receptors are overexpressed on cancerous cells, rendering normal cell expression of the receptors susceptible to inadvertent treatment by such nanoparticle combination therapy. Another approach which targets indirectly rather than on the cell surface involves targeting the source of cancer cell proliferation, cancer stem cells.

### 5.2. Indirect Cellular Targeting

Cancer stem cells (CSCs) are a promising new way to target the source of cancer development in the body [106]. Cancer stem cells have been used to describe the evolution of tumor cell heterogeneity, where cells of various cancer types mutate and adapt to survive in the tumor microenvironment by epigenetic changes, phenotypic and intratumoral heterogeneity, all working to promote cellular resistance to therapy [62]. CSCs are a novel target in research, as well as in clinical practice. DDS have been used to target the cell markers such as CD44, CD90, and aldehyde dehydrogenase, as well as signaling pathways like Notch and Hedgehog, that are characteristic of CSCs [121]. Cancer stem cells have an increased rate of target gene expression, alternative signaling pathway compensation mechanisms, and undergo dedifferentiation in an effort to evade destruction by drug therapy [121]. Cell quiescence, increased DNA repair, detoxifying enzymes, increased drug efflux, and higher levels of aldehyde dehydrogenase (ALDH) activity also encourage chemoresistance and tumorigenicity found in CSCs [121,122]. Li et al. dual encapsulated doxorubicin (DOX) and decitabine (NPDAC) in polymeric nanoparticles (MPEG-b-PLA) [123]. NPDAC can sensitize cells that have high ALDH to chemotherapy agents, such as DOX [123]. The results of this study showed that the combination delivery of these drugs in MDA-MB-231 breast cancer cells caused a reduced number of CSCs with increased levels of ALDH, in addition to overcoming drug resistance [123]. Cell marker, CD 44, is linked to CSC attributes such as tumor initiation, metastasis and chemo- and radio-resistance [35,124]. This cell marker was assessed and targeted in a study by Aires et al. which used functionalized iron oxide nanoparticles to encapsulate antiCD44 antibody and gemcitabine derivatives to treat CD44 positive pancreatic and breast cancer cell lines [35]. CD44 positive cells were selectively killed in this study using targeted treatment when compared to the control which was non-tumorigenic [35]. This study was additionally unique in how hyperthermia and contrast agents, using MRI, can be employed to thermally destroy cancer cells and visualize the effects of chemotherapeutics on CSCs [35].

Another study targeted breast cancer stem cells (bCSCs) by conjugating hyaluronic acid (HA) to polymeric, PLGA, nanoparticles which also dual encapsulated paclitaxel, a chemotherapeutic, and curcumin, a selective inhibitor of stem cells. The hyaluronic acid targeted CD44 receptors of breast cancer cells and led to decreased number and migration of bCSCs, as well synergistic growth inhibition of non-bCSCs and bCSCs in MCF7 xenograft tumor models [125]. Furthermore, another study which used hyaluronic acid to target CD44 found similar tumor inhibition and cytotoxicity in triple negative breast cancer cells by using microRNA (MiR-542-3p) and doxorubicin (DOX) combination loaded HA-conjugated polyethylenimine-poly(d,l-lactide-co-glycolide) (PEI-PLGA) nanoparticles [126].

Dually functionalized d-alpha-tocopheryl poly (ethylene glycol 1000) succinate (TPGS) and HA liposomes which were composed of 1,5-dioctadecyl-*N*-histidyl-l-glutamate (HG2C_18_), a synthetic cationic lipid, co-encapsulated paclitaxel (PTX) and lonidamine (LND). These liposomes were used to inhibit P-glycoprotein (P-gp) efflux and enhance mitochondrial drug accumulation in MDR breast cancer cells in vitro and MCF-7/MDR tumors in vivo. The HG2C_18_ lipid in particular demonstrated enhanced endo-lysosomal escape of the liposomes upon internalization by cells, thus promoting greater drug delivery and chemotherapeutic antitumor effects. The study found synergism between LND and PTX where LND sensitized cells by suppression of P-gp efflux, resulting in enhanced apoptotic effects of paclitaxel [127].

The understanding of how indirect cancer cell targeting can affect the tumor cell environment may be the link in providing more effective treatment, which stops the initiation of further tumor mutations at the stem cell level. Furthermore, the combination of direct and indirect targeting may prove more powerful, as we discuss next.

### 5.3. Dual Effect (Direct and Indirect) Cellular Targeting

**Figure 5:**
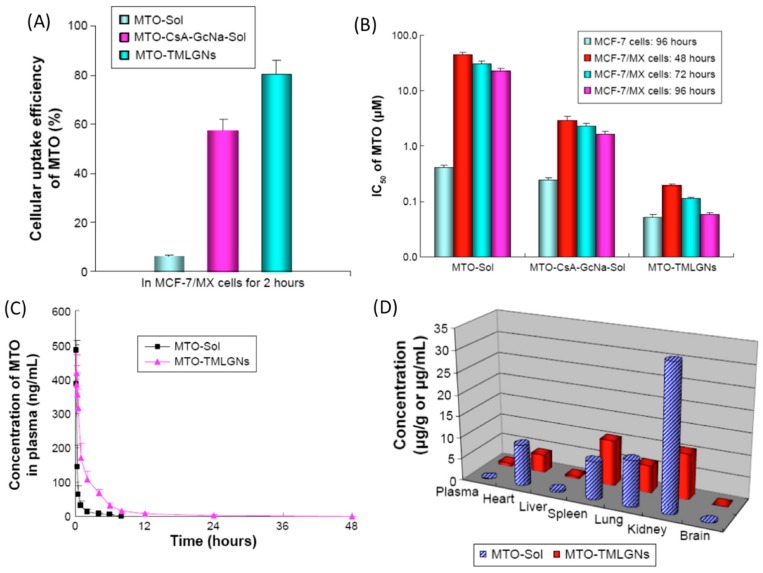
In vitro results for (**A**) cellular uptake and (**B**) cytotoxicity of various treatments of mitoxantrone hydrochloride (MTO), free drugs, MTO-Sol and MTO-CsA-GcNa-Sol and nanocarrier, MTO TMLGNs, formulations. The in vivo results show (**C**) plasma concentration differences between free drug MTO and nanocarrier form, where increased circulating drug concentration over time results from encapsulation, as well as (**D**) decreased overall organ accumulation of drug due to nanocarrier delivery of MTO, CsA and GcNa. Reproduced with permission from [59], Copyright Dovepress, 2015.

Dual effect cellular targeting, by combining direct and indirect cellular targeting modalities, may aid in the obstacle of multi drug resistance (MDR) encountered in cancer treatments [50,65,128,129,130,131,132,133]. Cellular resistance by both pump and non-pump mechanisms were investigated in a study done by Ling et al. which targeted drug efflux by P-glycoprotein and breast cancer resistance protein (BCRP) and immune response of anti-apoptosis by B cell lymphoma (Bcl-2) [134,135]. The goal of the study was to overcome MDR by dual cellular level targeting using a combination treatment of mitoxantrone hydrochloride (MTO), a water-soluble cation, cyclosporine A (CsA), a BCRP inhibitor, and sodium glycocholate (GcNa), a Bcl-2 inhibitor. Lipid-sodium glycocholate nanocarriers (TMLGNs) were used to encapsulate MTO, CsA and GcNa. BCRP and P-glycoprotein are overexpressed in cancer and contribute to MDR. MTO is a chemotherapeutic drug that has been used to treat advanced breast cancer, as well as prostate cancer, leukemia and lymphoma. Because MTO is a BCRP substrate, there is exists the issue of tumor cell resistance to this drug. CsA is a well-known inhibitor of BCRP and drug of choice for MDR-reversal, while GcNa is a Bcl-2 inhibitor that suppresses non-pump cellular resistance. MTO encapsulation in lipid nanocarriers is difficult due to its low molecular weight and hydrophilic characteristics, however this can be overcome by using a counterion, such as GcNa, in the TMLGNs which increases the encapsulation of MTO and sustains MTO release in the lipids. MCF-7, a human breast cancer cell line, and MCF-7/MX, a multidrug resistant variant which overexpresses BCRP, were used in this study to assess the cytotoxicity of MTO in the MCF/MX cells and MDR reversal of MTO. In vitro cytotoxicity studies indicated that TMLGNs increased delivery of MTO in cancer cells, as well as improved the sensitivity of cancer cells to the drug when compared to the free drug form of MTO and MTO-CsA-GcNa. The reversal factor (RF) and resistance index (RI) of MTO formulations were investigated and showed complete MDR reversal by MTO-TMLGNs where the RI was close to 1.0 and the RF values taken at 24, 48 and 96 h increased over time. MTO-TMLGNs were the most cytotoxic to cancer cells with the smallest IC_50_ value compared to other MTO drug formulations, as shown in Figure 5 as follows on the next page [59].

The pharmacokinetics and tissue distribution in vivo results showed that the free drug form of MTO (MTO-Sol) and nanoparticle form of MTO (MTO-TMLGNs) were significantly different regarding mean plasma concentration of MTO and relative tissue distribution. MTO-TMLGNs demonstrated a sustained plasma profile, as well as delayed systemic circulation retention and moderate biodistribution. The MTO-TMLGNs showed a 4.6-fold increase in the drug systematic exposure, in addition to a 7-fold increase in drug circulation time. MTO-TMLGNs also had a reduced distribution in organs such as the heart, kidneys, and lungs. The heart exposure to MTO was reduced by 0.6-fold, an important finding because of the risk of dose-related cardiomyopathy associated with the toxicity of MTO. There was a 12.8-fold higher uptake efficiency of MTO-TMLGNs in MCF/MX cells. These TMLGNs displayed both synergistic and complete reversal of MTO MDR. The encapsulation efficiency of MTO and promotion of cell death was achieved by using GcNA. Temperature and endocytosis inhibition experiments in MCF-7/MX cells showed that clathrin-mediated endocytosis was used by TMLGNs to enter cancer cells, therefore evading drug efflux by the BCRP transporter. CsA could then inhibit released MTO and overcome MDR by synergism with GcNA in this drug delivery model [59].

The co-encapsulation and delivery of a cationic hydrophilic antitumor drug, BCRP inhibitor, and Bcl-2 inhibitor was successfully achieved using TMLGNs in this study. The results demonstrated desirable pharmacokinetic release profile, uptake by clathrin-mediated endocytosis and cytotoxicity in resistant cancer cells with ultimate MDR reversal by drug synergism [59]. MDR is a prominent issue in effective cancer treatment, thus making relevant work critical to the future of practical treatment solutions for drug resistant cancer types [135,136,137].

## 6. Multi-tier (Vascular, Cellular and Tissue Combinations) Targeting

Simultaneously targeting various hallmarks of cancer poses as an effective strategy for cancer treatment, and in recent studies favoring the enhanced permeability and retention (EPR) effect, nanoparticles measuring less than 150 nm in size have exhibited effective co-delivery and multi-targeting abilities [35,124,128,129]. Moreover, multi-targeting at the vascular, tissue and cellular levels has been suggested as a competent approach [138,139,140,141]. Multi-tier targeting is any combination of vascular, cellular or tissue targeting methods, while the aforementioned dual effect cellular targeting combines direct and indirect cellular targeting specifically.

Nanoparticles have shown effective co-loading and a sustained release of several apoptosis inducers and anti-angiogenic agents along with peptide-conjugation which is made plausible by the flexibility in their physicochemical characteristics. In a study by Choi et al., a 120 nm-sized mesoporous silica nanoparticle (MSN) containing celastrol (CST) for mitochondrial blockade-mediated apoptosis and anti-angiogenic axitinib (AXT) in PEGylated lipid bilayer demonstrated multi-level targeting and synergistic effects in xenograft tumor mouse model. Targeting at the cellular and the vascular level, and given the association in their signaling pathways, the synergistic effect of these two therapeutic agents showed effective cell binding and sequential endocytotic uptake. A controlled and a pH-dependent release also emphasized the role of the tissue microenvironment in effective drug delivery [142]. Another formulation encapsulating paclitaxel (PTX) and VEGF targeting siRNA in a lipid nanoparticle shell conjugated with somatostatin-targeting peptide: Vapreotide (VAP) displayed a stable, sustained release and an efficient delivery to the tumor site. VAP-conjugated nanoparticles demonstrated a higher cellular uptake in MCF-7 cells as compared to their non-targeted counterparts, a result of rapid endosomal escape due to the effects of DOPE causing lysosome membrane disruption. Although in vivo nanoparticle accumulation was observed in organs of the reticuloendothelial system (RES) like liver for both targeted and non-targeted particles, the former showed higher PTX uptake in tumor areas [143]. MSN formulations measuring less than 100 nm conjugated with iRGD peptide also showed a collaborative co-loading of doxorubicin (DOX) for chemotherapy and combretastatin A4 (CA4) for anti-VEGF activity. The electrostatic interaction and hydrogen bonding between DOX and CA4 facilitated for a higher loading than their single-loaded counterparts. CA4 was released prior to DOX due to the strong electrostatic interaction between the negatively charged MSN and positively charged DOX which in this case coincidently proved to be favorable for this therapeutic approach. A higher uptake of targeted MSN in Hela cells was seen in this approach due to higher expression of a2b3 [144].

Targeted particles like VAP modified core-shell NPs have shown a higher VEGF-silencing activity and cell-cycle arrest in effect to chemotherapeutic drugs at certain concentrations while maintaining over 80% cell viability with similar effects in tumor mouse models [143]. Targeted liposomes too have shown successful translocation of encapsulated siRNA which otherwise incurs difficulties in delivery due to its poor membrane permeability. A formulation encapsulating docetaxel and VEGF siRNA conjugated with two different targeting peptides angiopep-2 and neuropilin demonstrated the most efficient uptake in vivo at an optimized conjugation ratio thereby showing a dual approach which assisted translocation with almost 20% higher VEGF silencing than controls in a U87 MG tumor-bearing mouse model [145].

**Figure 6:**
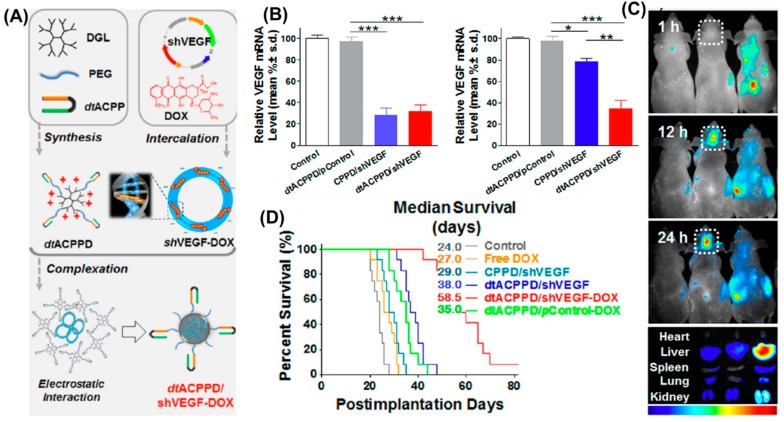
(**A**) Schematic showing a dtACPP-modified dendrigraft poly-l-lysine (DGL) nanoparticle system co-loading DOX intercalated into shVEGF. (**B**) Representation of in vitro (left) and in vivo (right) endogenous VEGF-silencing activity by different formulations. (**C**) In vivo distribution and localization of (L-R) non-targeted, dtACPP-modified and non-quenchable CPP DGL nanoparticles. The quenchable dtACPP particles show highest uptake in the brain. (**D**) Overall survival rates for glioma-bearing mice showing the highest rate for dtACPP-modified DGL particles. Reproduced with permission from [97], Copyright ACS, 2013.

In a study by Huang et al., the acidic property of the tumor tissue microenvironment was utilized in developing a pH-responsive DDS. A spherical and compact dendrigraft poly-l-lysine (DGL) nanoparticle system measuring approximately 150 nm in size with a zeta potential of 3.5 ± 2.9 mV was modified with pH-responsive cell-penetrating peptides (dtACPP). A masked form of this peptide was formulated to overcome CPP peptide’s poor specificity in vivo. These nanoparticles demonstrated that both matrix metalloproteinase 2 (MMP2) activity and an acidic pH (under 6.0) contributed directly to the cell-penetrating activity of masked dtACPP resulting in up to 86.9% internalization in U87-MG cells. Following passive targeting via the enhanced permeability and retention (EPR) effect, the dtACPP-modified nanoparticles actively targeted intracranial glioblastoma (GBM) xenograft mouse models. The loading of combination payload comprising of doxorubicin intercalated within a VEGF-targeting interfering RNA (shVEGF) was facilitated by the electrostatic interactions between negative and positive charges of shVEGF and DGL respectively. Highest in vivo localization of particles was observed in the tumor cytoplasm followed by nuclei, the latter being a significant finding for this application. Additionally, a reduction to 34.3% of endogenous VEGF mRNA and an apoptotic rate of 45.5% in vitro, and over twice the median survival rate of control in glioma-bearing mice were demonstrated by this DDS. This study effectively exhibits tumor regression by multi-level targeting at the tissue, vascular and cellular level, as shown in Figure 6 below [45].

## 7. Conclusions and Future Direction of Active Targeting

Nanomedicine has many advantages that shape its niche in modern cancer medicine. A multitude of desirable characteristics such as increased localized delivery of concentrated drug payloads, prolonged circulation in the bloodstream, reduced frequency of dosage required for therapeutic efficacy, uniform, sustained drug release kinetics, in addition to fewer systemic side effects and interactions, reduced vital organ accumulation and overall safety profile improvements in drug pharmacodynamics and pharmacokinetics create a lasting impression for nanotherapy in cancer treatment [31,74,146]. The value of therapeutic window expansion is most important in using nanoparticle drug delivery systems in an effort to provide cancer patients with safe, effective treatment, however the tumor microenvironment, physical attributes and evolving cellular pathways and adaptations present profound difficulties in reliable drug delivery and outcomes.

Several first-generation non-targeted nanomedicines have received clinical approval starting with Doxil^®^ in 1995. However, more than two decades on, targeted nanomedicines have not been approved yet, although several platforms have been in clinical trials. Patient to patient variability and tumor heterogeneity within the same patient present a major biological challenge to the design and development of targeted nanomedicines. Simultaneously targeting multiple biomarkers may therefore be required for efficacy. However, the complexity of design in targeted nanomedicines with the addition of one or more targeting ligands often can make them difficult to manufacture at large scales in a reproducible manner. A modular approach of assembling targeted nanomedicines may help scale up production of multi-targeted nanomedicines. Studies have also shown that targeting nanoparticles by surface functionalized antibody or peptide can sometimes lose its targeting capability upon adsorption of biomolecule corona or be detected by the MPS system, reducing circulation half-life and thereby hindering the efficacy of the targeted DDS [109].

Further, for targeted nanomedicines to be successful the cellular uptake and intracellular processing of the platform and the payload are vital. Most targeted nanoparticles use receptor mediated endocytosis as a mechanism for intracellular drug delivery [27]. Lysosomal degradation of the nanoparticles and the payload following receptor mediated endocytosis may be one of the reasons targeted nanomedicines have not shown the dramatic improvements in therapeutic index that was expected from these tumor homing constructs [147]. The development of novel targeting ligands (for e.g., peptides and aptamers) that can selectively trigger endosomal escape of the nanoparticles prior to lysosomal degradation may prove to be a promising strategy towards improving the therapeutic indices of targeted nanomedicines [42,139,148,149,150,151,152,153,154]. Overcoming intracellular and extracellular barriers is one of the major challenges in siRNA delivery. For example, Li et al. have developed humanized anti-EGFR mAb h-R3, a negatively charged ligand to surface functionalize binary complexes of self-assembled siRNA encapsulated poly(amidoamine), PAMAM. These dendriplexes exhibited remarkable endosomal escape in HepG2 cells and enhanced targeted drug delivery ex vivo [148].

Another hurdle in the development of targeted nanomedicines has been the lack of relevant pre-clinical models for testing their targeting efficiency. The development of more, relevant in vivo models that can recapitulate the complexity of human disease could help in optimizing the design of targeted nanomedicines during their pre-clinical development and perhaps ensure a higher percentage of success during clinical trials. Theoretical and mathematical models could be used to design better, more effective, targeted nanomedicines prior to pre-clinical and clinical testing. A multidisciplinary approach with collaborations between theoretical and experimental scientists, engineers, medical doctors, pharmaceutical and biotechnology industries, federal and private funding agencies and the regulatory agencies is therefore required to realize the true potential of targeted nanomedicines in the clinic.
